# Verrucomicrobia are prevalent in north-temperate freshwater lakes and display class-level preferences between lake habitats

**DOI:** 10.1371/journal.pone.0195112

**Published:** 2018-03-28

**Authors:** Edna Chiang, Marian L. Schmidt, Michelle A. Berry, Bopaiah A. Biddanda, Ashley Burtner, Thomas H. Johengen, Danna Palladino, Vincent J. Denef

**Affiliations:** 1 Department of Ecology and Evolutionary Biology, University of Michigan, Ann Arbor, MI, United States of America; 2 Annis Water Resources Institute, Grand Valley State University, Muskegon, MI, United States of America; 3 Cooperative Institute for Great Lakes Research, University of Michigan, Ann Arbor, MI, United States of America; Wageningen University, NETHERLANDS

## Abstract

The bacterial phylum Verrucomicrobia was formally described two decades ago and originally believed to be a minor member of many ecosystems; however, it is now recognized as ubiquitous and abundant in both soil and aquatic systems. Nevertheless, knowledge of the drivers of its relative abundance and within-phylum habitat preferences remains sparse, especially in lake systems. Here, we documented the distribution of Verrucomicrobia in 12 inland lakes in Southeastern Michigan, a Laurentian Great Lake (Lake Michigan), and a freshwater estuary, which span a gradient in lake sizes, depths, residence times, and trophic states. A wide range of physical and geochemical parameters was covered by sampling seasonally from the surface and bottom of each lake, and by separating samples into particle-associated and free-living fractions. On average, Verrucomicrobia was the 4^th^ most abundant phylum (range 1.7–41.7%). Fraction, season, station, and depth explained up to 70% of the variance in Verrucomicrobia community composition and preference for these habitats was phylogenetically conserved at the class-level. When relative abundance was linearly modeled against environmental data, Verrucomicrobia and non-Verrucomicrobia bacterial community composition correlated to similar quantitative environmental parameters, although there were lake system-dependent differences and > 55% of the variance remained unexplained. A majority of the phylum exhibited preference for the particle-associated fraction and two classes (Opitutae and Verrucomicrobiae) were identified to be more abundant during the spring season. This study highlights the high relative abundance of Verrucomicrobia in north temperate lake systems and expands insights into drivers of within-phylum habitat preferences of the Verrucomicrobia.

## Introduction

The bacterial phylum Verrucomicrobia was first observed in 1933 in freshwater aquariums [1, 2] and later isolated from a freshwater pond in 1970 [3] However, it was not until 1997 that it was officially recognized as a phylum [4] Named after the verruca-like, or pedal wart-like, appearance of one of its members, *Verrucomicrobium spinosum* [5], this phylum has been found in various environments around the world, including soils [6–9], oceans [10, 11], freshwater lakes [12–14], and gastrointestinal tracts [15, 16].

The abundance of Verrucomicrobia has been largely overlooked in the past, likely due to primer bias as common PCR primers fail to target this phylum [7], as well as large DNA extraction efficiency variation between protocols for the phylum [17]. In soils, Verrucomicrobia was previously reported to constitute an average of 7% of the bacterial community [6]. However, with the use of improved primers, it was observed that Verrucomicrobia accounted for an average of 23% of the bacterial community and was found in 180 out of 181 soil samples, with the highest relative abundance (35%) in grasslands and subsoil [7].

Based on 16S rRNA phylogeny, Verrucomicrobia has been categorized into seven subdivisions [18]. Spartobacteria (subdivision 2) is the predominant verrucomicrobial class in soils [6, 7, 19] and has been detected in varying levels in aquatic environments [11, 20]. Members of this class were identified to contain diverse carbohydrate-degrading enzymes, as well as a full sulfate utilization pathway [21]. Verrucomicrobiae (subdivision 1) and Opitutae (subdivision 4) have been found to comprise large proportions of the verrucomicrobial community in marine systems (31% and 40% in water; 57% and 27% in sediment, respectively) [11]. Nitrogen fixation genes were identified in some Opitutae members [22]. Subdivision 5 is characterized by members found in anoxic environments and has been proposed to be categorized as the novel phylum Kiritimatiellaeota [23]. Subdivisions 3, 6, and 7 remain uncultured. Subdivision 3 comprises a large proportion of verrucomicrobial soil communities and contains the class OPB35 soil group [24, 25]. Members of subdivision 6 include aerobic methanotrophs, the first discovered outside of Proteobacteria [26], some of which are capable of fixing nitrogen [27]. While Verrucomicrobia is extremely prevalent, has diverse metabolic capabilities, and possesses great potential to play important roles in various global geochemical cycles, little is known about its ecology in freshwater systems. In particular, which physical and geochemical drivers determine this phylum’s relative abundance and community composition, and consequently the nature of within-phylum habitat differentiation, remain unclear. Yet, freshwater systems have recently been recognized as hotspots for carbon cycling, contributing significantly to global carbon fluxes [28–30].

In this study, 16S rRNA gene sequencing of the V4 hypervariable region was used to analyze the distribution and diversity of Verrucomicrobia in 14 different north temperate freshwater lakes. Given the phylum’s diverse metabolism, we expected to see distinct relative abundances and community compositions in different lake environments. We examined the correlation between the phylum’s relative abundance and various geochemical variables, and analyzed categorical variables that explained the within-phylum composition variation. Compositions of Verrucomicrobia and non-Verrucomicrobia bacterial communities were correlated to environmental parameters to evaluate whether similar parameters drove within-Verrucomicrobia and non-Verrucomicrobia bacterial community composition. Our study highlights the high relative abundance of Verrucomicrobia in freshwater lakes and provides insights into its within-phylum habitat preferences.

## Results

### Survey description

**Fig 1:**
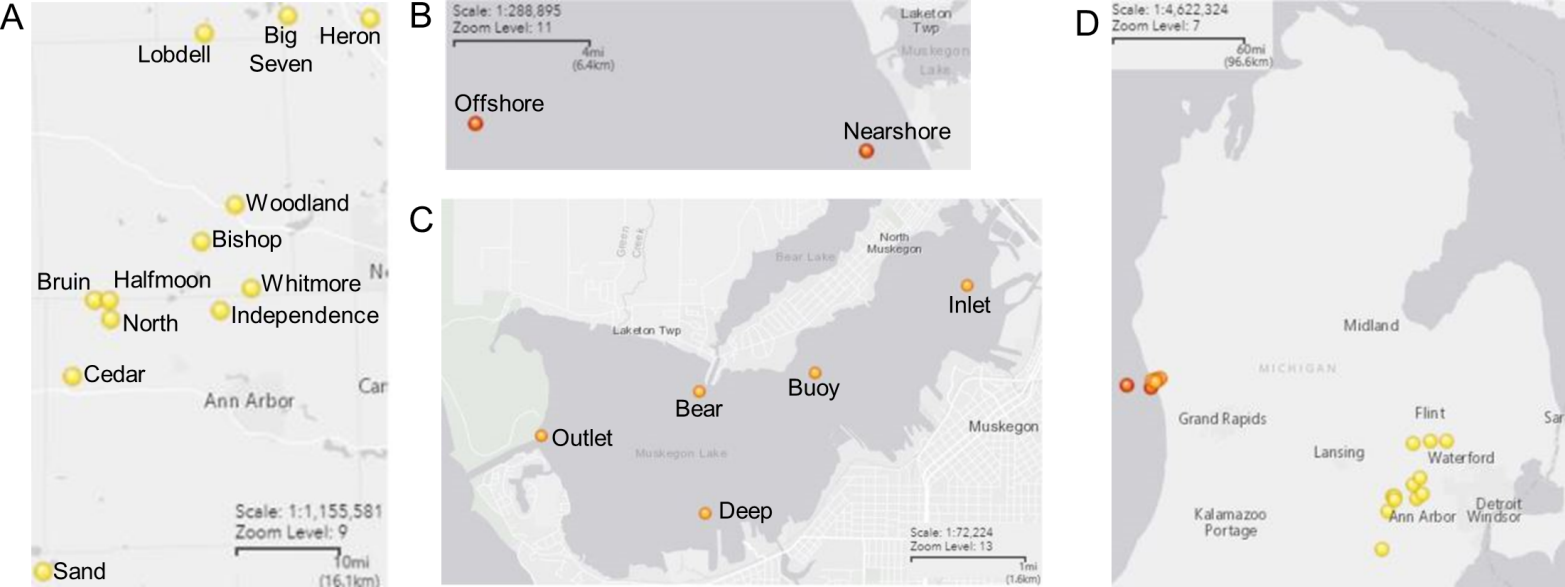
Maps of sampling site locations. (A) Inland lakes (yellow), (B) Lake Michigan (red), and (C) Muskegon Lake (orange), with (D) the locations displayed together. Map created using the U.S. Geological Survey, National Geospatial Program, National Map Viewer.

Free-living (FL; 0.22–3 μm) and particle-associated (PA; 3–20 μm)) water bacterial community samples were collected in spring, summer, and fall from (a) twelve southeastern Michigan lakes (referred to as “Inland” below), (b) from a near to offshore transect in Lake Michigan, a Laurentian Great Lake (“Laurentian”), and (c) from Muskegon Lake, a drowned river mouth freshwater estuary of Lake Michigan (“Estuary”) (Fig 1); sediment samples were also collected from Muskegon Lake. After combining data from biological replicates, we successfully generated 228 sequencing data sets of the V4 region of the 16S rRNA gene (mean = 26,850 reads per sample, range = 1–439,926 reads per sample). We removed 2 samples that resulted in fewer than 2,000 reads from our analysis and normalized samples by scaling to the smallest library size (2,072 sequences) and rounding counts (the approach developed by McMurdie and Holmes (see methods section)). After normalization, the range of reads in our samples was 1,425–2,096 reads. Environmental data that we collected at the time of sampling indicated that we sampled across a wide range of temperatures (2.71°C—24°C), and lake trophic states (Chl *a* from 0 to 8.96 ug/L and TP from 2.55 to 48.7 ug/L; S1 Table). While the same sampling scheme was followed in all lakes (surface vs. bottom; spring vs. summer vs. fall; FL vs. PA), different sets of environmental data were available from each system (Inland, Laurentian, Estuary) based on system-dependent standard operating procedures from our different collaborators. Hence, we performed analyses of community patterns and correlation to quantitative environmental parameters for each system separately.

### Comparison of samples from Laurentian and estuary surveys

To assess bias due to differences in the primer tails used by the two sequencing centers, we compared Verrucomicrobia relative abundance between samples from the Muskegon Lake buoy site taken on the Laurentian survey (Joint Genome Institute) and other Muskegon Lake samples taken on the estuary survey (University of Michigan, same as inland survey). For samples collected during the Laurentian survey, the offshore Lake Michigan station had significantly less Verrucomicrobia compared to the nearshore Lake Michigan and the Muskegon Lake buoy stations (Kruskal-Wallis (KW), p-values = 0.018 and 0.021, respectively; S1A Fig). Among water samples taken from Muskegon Lake during both surveys, there was only a significant difference in Verrucomicrobia relative abundance between the deep station (estuary survey) and the buoy site (Laurentian survey) (KW, p-value = 0.019; S1B Fig); however, this result was caused by an outlier in the deep station. After removing the outlier, the two stations were not significantly different. These data suggest the effect of sequencing center was limited. However, the environmental data available for the buoy site different from that available for the other Muskegon Lake stations as these sites were examined on different surveys. Therefore, buoy samples from the Laurentian survey were removed from further analyses to avoid introducing potential bias into the analysis of environmental drivers of Verrucomicrobia abundance and composition in estuary samples.

### Verrucomicrobia relative abundance, within-phylum diversity, and phylogenetic diversity

Verrucomicrobia was the 4th most abundant phylum, with a median relative abundance across all samples of 9.3% (range: 1.7–41.7%; S2 Fig). Verrucomicrobia relative abundance was significantly lower in Laurentian samples (4.7 ± 3.0 (interquartile range) %) compared to estuary (11.0 ± 9.4%) and inland samples (10.3 ± 8.5%) (Kruskal-Wallis, p-value < 0.001).

**Fig 2:**
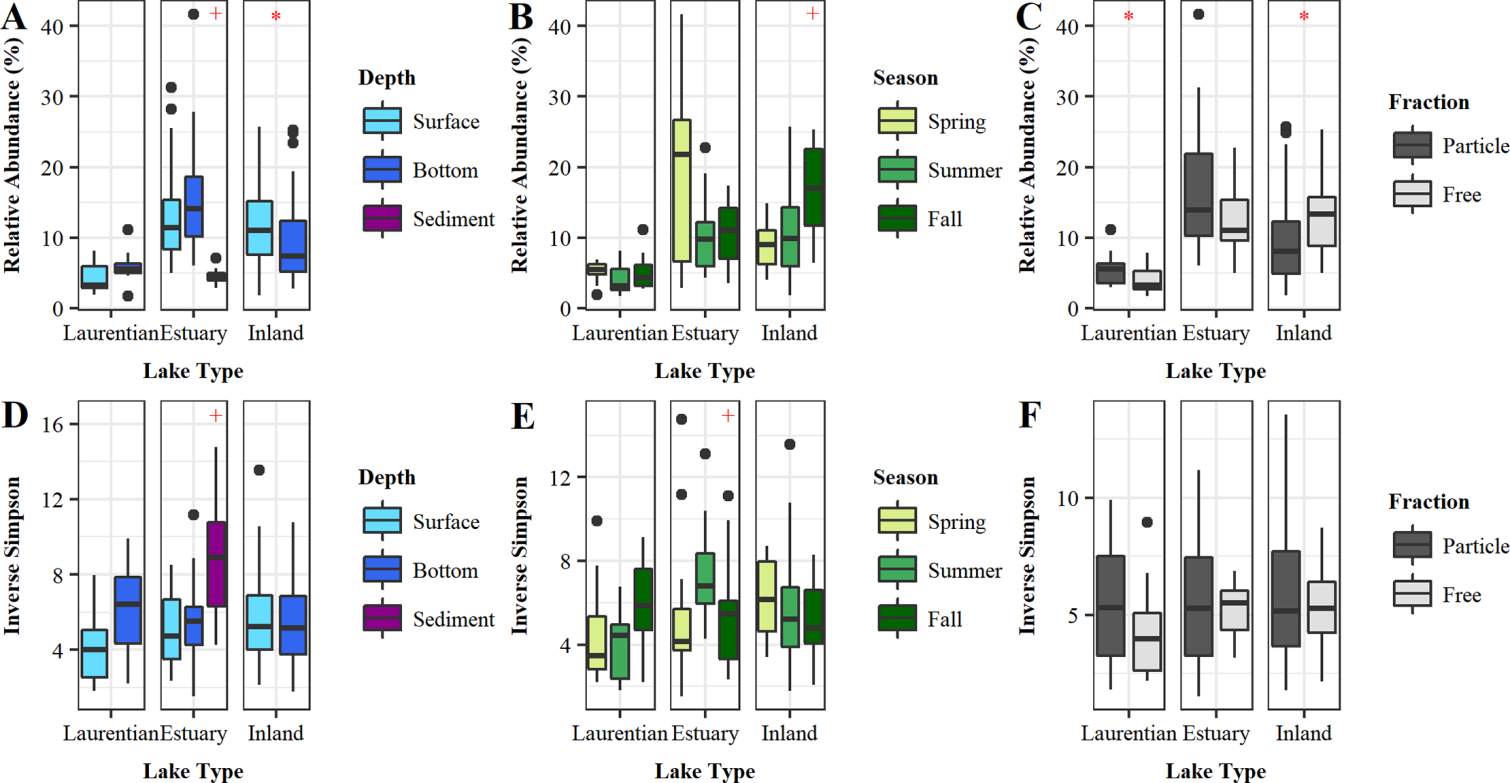
Verrucomicrobia relative abundance box-and-whisker plot and inverse Simpson. Verrucomicrobia relative abundance is illustrated in samples categorized by lake type and (A) sampling depth, (B) season, or (C) fraction. Inverse Simpson averaged from 100 trials is illustrated in samples categorized by lake type and (D) sampling depth, (E) season, or (F) fraction. Red asterisks indicate significance between all samples in the panel. Red plus signs indicate significance of one subset of samples within the panel.

There was no significant difference between surface and bottom samples among Laurentian and estuary samples. Inland samples, however, had a significantly higher relative abundance in surface samples compared to bottom samples (KW, p-value = 0.033; Fig 2A). Similarly, among Laurentian and estuary samples, there was no significant seasonal difference in relative abundance (Fig 2B). However, among inland lake samples, the Verrucomicrobia relative abundance was significantly higher in fall compared to spring and summer (KW, p-value = 0.001; Fig 2B). The Verrucomicrobia relative abundance in PA samples was significantly higher compared to FL samples in Laurentian samples, while the opposite was true for inland samples (KW, p-values = 0.028 and < 0.001, respectively; Fig 2C). In the estuary, sediment samples had a significantly lower relative abundance compared to water samples (KW, p-value < 0.001; Fig 2C).

To evaluate the relationship between Verrucomicrobia relative abundance and geochemical factors, we used the quantitative environmental data available for each lake type to build the best multiple linear regression model. Only for the estuary did the available data allow us to construct a multiple linear regression model with a high correlation coefficient (R^2^ = 0.60; Table 1; S3 Fig). Temperature was weighted the most in the model and indicated a negative relationship between increasing temperatures and Verrucomicrobia relative abundance (coefficient = -0.97 when assessed individually; p-value = 0.002, adjusted R^2^ = 0.27).

**Table 1:** Multiple linear regression model for Verrucomicrobia relative abundance.

	Tested Parameters	Best Parameter(s)	Coefficient	Adjusted R^2^
**Laurentian**	DO	SiO_2_	1.190 (0.13)	0.062
	SiO_2_			
	Temperature			
**Estuary**	Chl *a*	ORP	0.036 (0.01**)	0.5989
	ORP	Temperature	-0.564 (0.008**)	
	SO_4_^2-^	TDS	-0.111 (<0.001***)	
	TDS			
	Temperature			
**Inland**	Chl *a*	NH4	-0.007 (<0.001***)	0.06124
	NH_4_			
	SRP			

Environmental variables and Verrucomicrobia were log-transformed to improve linearity when plotted against each other. Only variables that exhibited linearity and were not co-correlated with other included parameters were tested. Chl *a* = Chlorophyll *a*, DO = dissolved oxygen, NH_4_ = ammonium, ORP = oxidation-reduction potential, SiO_2_ = silica, SO_4_^2-^ = sulfate, SRP = soluble reactive phosphorus, TDS = total dissolved solids.

** P ≤ 0.01

*** P ≤ 0.001

Given the observed differences in relative abundance, we examined if this also translated into differences in Verrucomicrobia within-phylum diversity, measured by the inverse Simpson index, and phylogenetic diversity, measured by standardized effect size mean pairwise distance (SES MPD). Sediment samples harbored more diverse Verrucomicrobia communities than water column samples (KW, p-value = 0.01, Fig 2D), and estuary summer samples were significantly less diverse than both estuary spring and fall samples (KW, p-values = 0.011, Fig 2E). No significant differences were detected in Verrucomicrobia diversity across lake types, between PA and FL samples, across seasons, and between surface and bottom samples.

Although all lake types had positive SES MPD values (S4A Fig), indicating phylogenetic evenness, estuary samples had a large range (-3.2 to 2.1) due to sediment samples being more phylogenetically clustered than water samples (KW, p-value < 0.001; S4B Fig). When only considering water samples, SES MPD was significantly different between all lake types (KW, p-value < 0.001; S4A Fig). Differences in phylogenetic diversity between fraction and season depended on the lake survey (S4B and S4C Fig), while there was no significant difference between surface and bottom samples.

### Compositional differences in Verrucomicrobia and total bacterial communities

Both Verrucomicrobia and non-Verrucomicrobia community compositions were a function of lake type (PERMANOVA, Verrucomicrobia: R^2^ = 0.11, p-value = 0.001; non-Verrucomicrobia: R^2^ = 0.10, p-value = 0.001). While we focused on the Verrucomicrobia communities, we assessed whether similar factors drive Verrucomicrobia relative to non-Verrucomicrobia community composition shifts. Procrustes analyses were used to evaluate the correlation between the differences between samples in Verrucomicrobia and non-Verrucomicrobia communities. Estuary Verrucomicrobia and non-Verrucomicrobia bacterial community ordinations were the most strongly correlated (Procrustes, p-value = 0.001, correlation = 0.96), followed by Laurentian (p-value = 0.001, correlation = 0.80) and inland (p-value = 0.001, correlation = 0.77).

**Fig 3:**
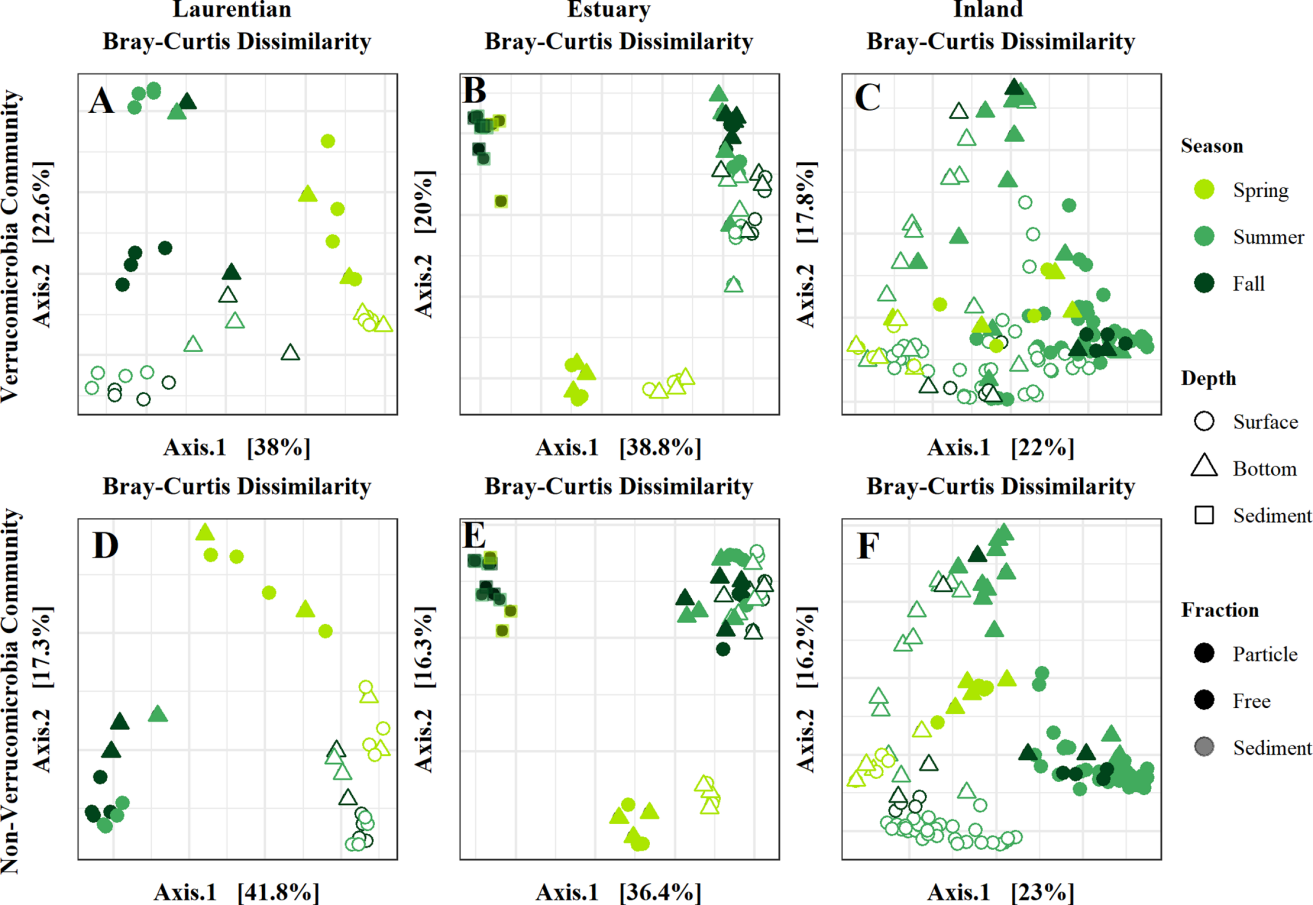
Principal coordinates analysis (PCoA) ordinations (first two principal coordinates are displayed) based on Bray-Curtis dissimilarity. PCoAs visualizing the compositional differences of **(A-C)** the verrucomicrobial and **(D-F)** the whole bacterial community in Laurentian, estuary, and inland lake samples, respectively. Data points are colored by season, shaped by depth, and filled in by fraction. Axis labels include the % variation captured by the respective dimension of the ordination.

In Laurentian samples, approximately 60% of the verrucomicrobial and non-verrucomicrobial community compositional variation was explained by season and fraction. Season was by far the most important factor for Verrucomicrobia, while fraction was the more important factor for the remainder bacterial community (Table 2). This also became apparent when representing the data on a PCoA, as spring samples clustered separately from summer and fall samples, and FL and PA fractions formed separate clusters as well (Fig 3A and 3D).

**Table 2:** Factorial variables correlating with variation in Verrucomicrobia and non-Verrucomicrobia communities (Nested PERMANOVA).

	Laurentian (n = 35)		Estuary (n = 55)		Inland (n = 126)	
	VER	NON-VER	VER	NON-VER	VER	NON-VER
**Source**	NA	NA	0.234 (0.001***)	0.333 (0.001***)	NA	NA
**Fraction**	0.173 (0.001***)	0.350 (0.001***)	0.132 (0.001***)	0.116 (0.001***)	0.112 (0.001***)	0.180 (0.001***)
**Season**	0.433 (0.001***)	0.234 (0.001***)	0.215 (0.001***)	0.175 (0.001***)	0.077 (0.001***)	0.100 (0.001***)
**Station**	0.035 (0.013*)	0.044 (0.012*)	0.036 (0.024*)	0.056 (0.001***)	0.271 (0.001***)	0.202 (0.001***)
**Depth**	0.045 (0.003**)	0.033 (0.022*)	0.015 (0.038*)	0.018 (0.018*)	0.077 (0.001***)	0.098 (0.001***)
**Residuals**	0.314	0.339	0.268	0.302	0.461	0.422

Station refers to different sites in Lake Michigan and Muskegon Lake for Laurentian and estuary samples (respectively). For inland samples, station refers to the 12 different inland lakes. R^2^ values are presented with the corresponding p-values in parentheses.

* P ≤ 0.05

** P ≤ 0.01

*** P ≤ 0.001

In the estuary, approximately 50% of compositional variation in Verrucomicrobia and non-Verrucomicrobia communities was explained by source (sediment vs. water samples) and season (Table 2; Fig 3B and 3E). Sampling depth was also important for variation in the non-Verrucomicrobia, but only correlated weakly with shifts in Verrucomicrobia community composition (Table 2). Fraction and spatial heterogeneity (Station) within the estuary accounted for similar levels of compositional variation between non-Verrucomicrobia and Verrucomicrobia communities (Table 2).

In inland lakes, variation in verrucomicrobial and non-verrucomicrobial communities was explained by the same factors, though the order of importance was different and the amount of total variation explained was lower than that of estuary and Laurentian samples (Table 2). The specific lake from which the samples originated (Station) accounted for the most variation. In contrast to the two other lake types, season explained much less variation, and variation between station obscured the seasonal patterns on the first two axes of the PCoA (Fig 3C and 3F). Similar to estuary and Laurentian lakes, variation explained by depth was limited (Table 2).

To explore which physicochemical parameters correlated with shifts in community composition, we performed a bioenv analysis, which identified different factors to affect non-Verrucomicrobia and Verrucomicrobia community composition in Lake Michigan (Table 3). The environmental factors measured in the estuary did not significantly correlate with either community’s composition (Table 3). In the inland lakes, nitrogen and phosphorus levels were included in the best predictors of both non-Verrucomicrobia and Verrucomicrobia community composition, although this was not significant for the non-verrucomicrobial community (Table 3).

**Table 3:** Continuous variables correlating with variation in the composition of Verrucomicrobia and non-Verrucomicrobia communities (Bioenv).

	Laurentian (n = 24)		Estuary (n = 44)		Inland (n = 126)	
	VER	NON-VER	VER	NON-VER	VER	NON-VER
**Tested parameters**	Chl *a*, DO, Fluorescence, DOC, PAR, POC, PON, PP, SiO_2_, TDP, Temperature, TP, TSS		Alkalinity, BGA, Chl *a*, Cl, DO, NH_3_, NO_3_^-^, ORP, pH, SO_4_^2-^, SRP, TDS, Temperature, TKN, TP, Turbidity		Chl *a*, NH_4_^+^, NO_3_^-^, SRP, TDP, TP	
**Best parameter(s)**	DO	TDP	NH_4_^+^	NH_4_^+^	NO_3_^-^	NO_3_^-^
		TSS	SRP	SRP	SRP	TDP
		PAR	DO	DO	TP	
					NH_4_^+^	
**Correlation**	0.443 (0.01**)	0.373 (0.02*)	0.125 (0.69)	0.189 (0.39)	0.445 (0.01*)	0.063 (0.4)

BGA = blue-green algae, Chl *a* = Chlorophyll *a*, DO = dissolved oxygen, DOC = dissolved organic carbon, NH_3_ = ammonia, NH_4_^+^ = ammonium, NO_3_^-^ = nitrate, ORP = oxidation-reduction potential, PAR = photosynthetically active radiation, POC = particulate organic carbon, PON = particulate organic nitrogen, PP = particulate phosphorus, SiO_2_ = silica, SO_4_^2-^ = sulfate, SRP = soluble reactive phosphorus, TDP = total dissolved phosphorus, TKN = total Kjeldahl nitrogen, TP = total phosphorus, TSS = total suspended solids.

* P ≤ 0.05

** P ≤ 0.01

### Class-level lake habitat specialization within the Verrucomicrobia

Considering the differences in phylogenetic diversity when comparing the Verrucomicrobia community across samples, and the apparent shifts in community composition highlighted above, we sought to determine whether there was a phylogenetic signal in habitat specialization. As a first analysis to determine whether Verrucomicrobia communities were phylogenetically distinct in association with the habitat from which they were sampled, we compared communities by using a weighted UniFrac distance metric. We found that lake type, fraction, season, and depth all contributed significantly to partition phylogenetically-weighted community composition variance (nested PERMANOVA, all p-values = 0.001, R^2^ = 0.15, 0.14, 0.07, and 0.04, respectively). To more clearly determine habitat partitioning between Verrucomicrobia clades, we mapped differential representation across habitat (depth, lake type, fraction, and season) onto a phylogenetic tree. We analyzed the class level as it was the most resolved taxonomic level at which the majority of Verrucomicrobia OTUs were classified (93.4%; S5 Fig).

**Fig 4:**
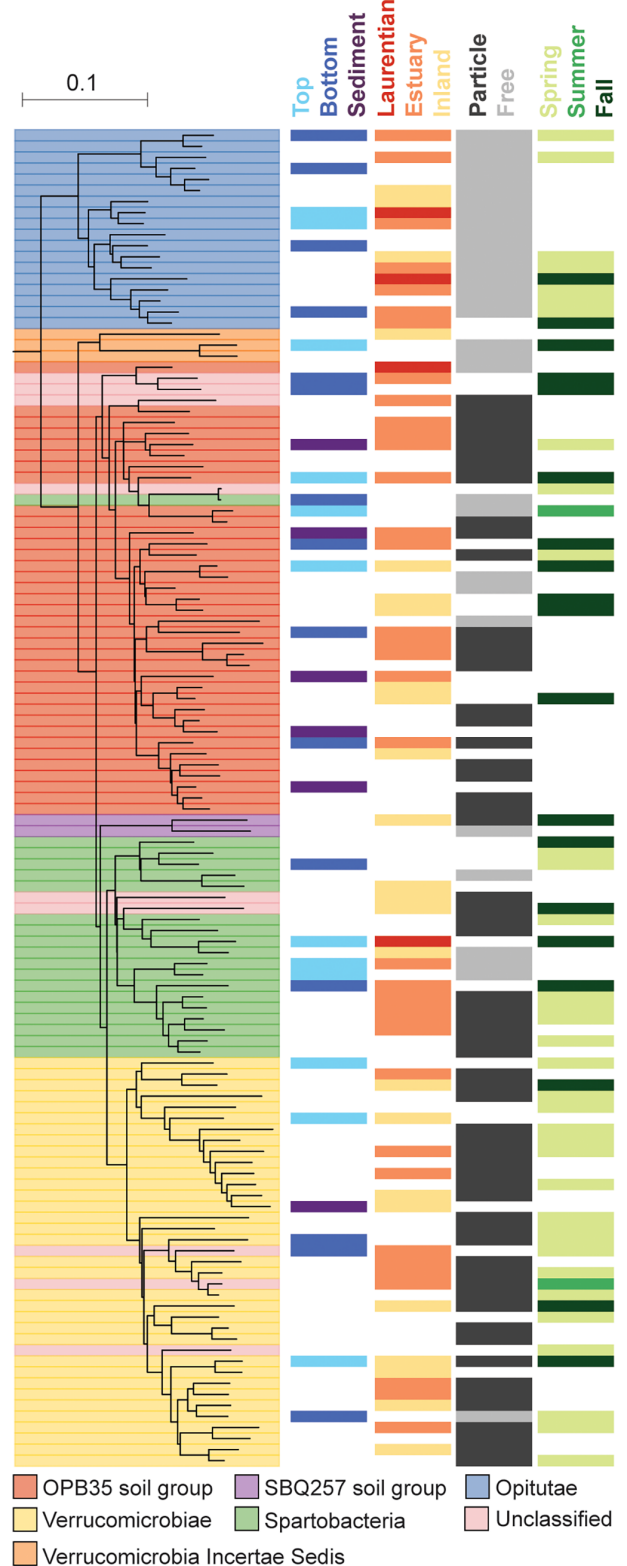
Verrucomicrobia phylogenetic tree and OTU habitat preferences. Only OTUs with a significant preference in at least one habitat were displayed. Habitats were categorized based on depth (surface, bottom, sediment), lake type (Laurentian, estuary, inland), fraction (particle, free), and season (spring, summer, fall). The tree is colored by verrucomicrobial class.

At the class level, which was the most resolved level of classification for 44% of Verrucomicrobia OTUs (S5 Fig), differences in relative abundance became apparent when plotting relative abundances across lake types, fractions, and seasons (S6 Fig). Opitutae was shown to be present at consistently higher relative abundance in the FL fraction, while other classes had higher relative abundance in PA fraction (Fig 4 and S5 Fig). As was indicated by their higher overall relative abundance in the estuary (S6 Fig), the OTU-level analyses also indicated significant overrepresentation of OPB35 soil group class OTUs within the Verrucomicrobia communities in the estuary, and a preference for fall (Fig 4). In contrast, the Verrucomicrobiae class showed a preference for spring. As measured by Pagel’s λ, lake type, fraction, and season had significant phylogenetic signals (p-values = 0.027, < 0.001, < 0.001 respectively) while sampling depth did not have a significant phylogenetic signal (p-value = 0.161).

## Discussion

Our results emphasize the numerical importance of Verrucomicrobia in a series of north-temperate lakes and reveal class-level preferences for lake habitats defined by filter pore size fraction, season, and lake type. Due to methodological biases, Verrucomicrobia have remained under-detected despite being one of the most abundant phyla across both aquatic and terrestrial ecosystems. One of these biases include sequencing primers that underestimated their relative abundance in both freshwater [31] and soils [6, 7]. When Newton and co-authors compiled a thorough review of sequencing-based freshwater microbial diversity assessments in 2011 [31], Verrucomicrobia was described as a relatively low abundance phylum, comprising between < 1% and 6% of sequences. However, use of an optimized fluorescent *in situ* hybridization (FISH) probe indicated true abundance may be much higher, with proportions of Verrucomicrobia observed up to 20% [20]. More recent studies that use primers targeting hypervariable regions of the 16S rRNA gene have also led to increased detection levels for Verrucomicrobia, both in soils [7, 32] and aquatic samples [33]. The V4 region primers we used covered 84.1% of the Verrucomicrobia sequences in the Silva reference database and have been found to potentially be biased for the phylum (5.9% versus 3.2% relative abundance inferred from amplicon and shotgun data, respectively) [9]

In addition to primer bias, we previously showed that, depending on the DNA extraction method, Verrucomicrobia detection levels in the same water sample ranged between 1.4% and 12.1% of the community [17]. The protocol used in the current study is the one that resulted in higher Verrucomicrobia detection, which McCarthy and co-authors validated by performing FISH using samples from the same freshwater estuary studied here [17]. As such, the numbers reported here are higher compared to those from sequencing surveys of north-temperate lakes summarized by Newton and co-authors (2–42% vs <1–6%; [31]). Despite potential biases introduced by primers and DNA extraction method, our previously shown correspondence between the 16S rRNA gene sequencing and microscopy data make us confident that our numbers provide a reliable estimate of the distribution and abundance of this phylum across the sampled lakes.

In freshwater estuary sediment samples, we found relatively low Verrucomicrobia abundance, which is contrary to findings in marine sediment [34]. This difference may be due to environmental differences, as previous studies have found the relative abundance of marine sediment Verrucomicrobia to increase with salinity [34], or due to differences in both extraction and sequencing methods. We also found that freshwater sediment Verrucomicrobia relative abundance was lower than that of adjacent water samples, although this may in part be due to extraction kit differences, as we used a MoBio kit for sediment samples to avoid interference by humic acids in the sediment. The higher diversity of Verrucomicrobia in sediment compared to water is in line with community-level diversity differences between water column and sediment bacteria [35]. The predominance of the OPB35 soil group (subdivision 3), which has been shown to be abundant in wetland soils [36], among sediment Verrucomicrobia is noteworthy and likely contributes to increased phylogenetic clustering in sediment samples.

The amount of variance in Verrucomicrobia relative abundance and community composition that was explained by our factorial and physical/geochemical parameters was relatively low. This indicates that factors beyond the ones we measured may be key in determining these characteristics. For example, the concentration and type of organic carbon available, which were not measured in our study, may play an important role in shaping verrucomicrobial community composition. A recent metagenomic study found Verrucomicrobia in a dystrophic bog to be more abundant and persistent, and to contain glycoside hydrolases specialized for different carbon substrates than those in a eutrophic lake [37]. In addition to geochemical factors, biotic interactions between Verrucomicrobia, other organisms, and viruses were not determined here and can influence the phylum’s relative abundance and composition. For example, marine Verrucomicrobia have been found to increase in relative abundance prior to cyanobacterial blooms and decrease post-bloom [38]. Specifically, ‘*Candidatus* Spartobacteria baltica’ was found to increase by tenfold prior to a cyanobacterial bloom and peaked twice post-bloom [38]. Furthermore, Verrucomicrobia have also been found to prefer high molecular weight dissolved organic matter, often produced by phytoplankton [21]. Additional variables must be considered in order to identify key environmental characteristics that influence Verrucomicrobia community dynamics.

We found variable levels of correspondence between our results and previously identified drivers of Verrucomicrobia abundance. While Lindström and co-authors documented increases in relative abundance with lake hydraulic retention time (9–730 days) and temperature (3–20.5°C) [39], the lake with highest retention time (Lake Michigan, 62 years) in our study had the lowest Verrucomicrobia levels, while the lake with the lowest retention time (Muskegon Lake, average of 21 days [40]) had the highest Verrucomicrobia levels. When temperature was identified as a significant factor in the multiple linear regression model, the relationship was opposite of findings in Swedish inland lakes [41]. It must be noted that Lindström and co-authors used a probe that targeted a subset of the Optitutae class only. A more resolved look at our data indeed showed that Opitutae can be more abundant in Lake Michigan depending on the season and fraction; however, their relative abundance also declines as the season progresses and temperatures increase. Temperature therefore may not be a causal factor, but instead co-correlate with other parameters that were not measured in our or the Lindström study. Increased phosphorus and nitrogen levels have also been linked to both increases [41–43] and decreases in Verrucomicrobia relative abundance [20]. While we did not identify P as a significant factor in our multiple linear regression modeling of Verrucomicrobia relative abundance, a weak negative relationship with ammonia levels was observed in the inland lake dataset. The discrepancies between our and previous findings, while in part due to mismatches in methodologies, are also highlighted by the divergent results from the multiple regression models by lake type in our study. As clades within the phylum may respond differently to environmental gradients [31, 39], we focused more on within-phylum diversity and habitat preferences for the remainder of our study.

Verrucomicrobia community composition shifts were often in line with those of the non-Verrucomicrobia community, although differences based on lake type emerged. Dissolved oxygen was a driver of Verrucomicrobia community composition, but did not influence the phylum’s relative abundance. Similar to previous findings of the importance of nutrient levels for specific groups of Verrucomicrobia, both P and N levels were identified as geochemical parameters correlated to the variation in Verrucomicrobia community composition. However, the strongest habitat preference was observed between the particle-associated (PA) and free-living (FL) fractions, which was consistent across all lake systems and seasons for the Opitutae (FL) and Verrucomicrobiae (PA). Class-level habitat preferences between PA and FL fractions have been observed across the bacterial domain in both freshwater [44] and marine systems [45], and suggest deep phylogenetic trait conservation to enable a particle-associated lifestyle. Such deep phylogenetic conservation is in contrast with other reports that highlight strain-level partitioning based on seasonality and particulate and free-living habitats [46]. It has to be some OTUs diverged from the predominant class-level pattern, suggesting habitat partitioning between PA and FL habitats occurs within-class as well, though preference of most OTUs within a class was for either the PA or FL habitat.

Based on our and previous results, factors that may shape Verrucomicrobia community composition include temperature, which has been shown to strongly affect habitat partitioning among bacterial groups even at the sub-species level [47], as well as season- and system-dependent inputs of DOC and POC. This includes responses to phytoplankton blooms, as noted above, which are correlated to both temperature changes and changes in DOC/POC composition. C source quality and quantity may be key in shaping Verrucomicrobia community composition, and additional studies are needed to examine this relationship. Enzymatic assays and inferences from genomic analyses, including from genomes reconstructed from freshwater and marine water and sediment metagenomic data, have indeed tied Verrucomicrobia to the metabolism of diverse carbon substrates such as laminarin, xylan, mannan, chitin, cellulose, and starch [33, 48]. In addition to glycoside hydrolases [21, 37, 49], Verrucomicrobia also contain genes for many carbohydrate esterases and some extracellular peptidases [49]. Not only do Verrucomicrobia harbor a diverse set of carbohydrate metabolism enzyme-encoding genes, but they have been found to contain more of such genes than any other bacterial group in the community [48, 49]. A recent freshwater metagenomic study of 19 Verrucomicrobia draft genomes also identified sulfatases, which degrade sulfated polysaccharides, as well as various genes involved in carbohydrate transport across both outer and inner membranes, suggesting that members of this phylum act as polysaccharide degraders in freshwater systems [37]. These findings all highlight Verrucomicrobia’s potential role in carbon cycling in aquatic environments.

In conclusion, despite initial recognition as a low abundance group, Verrucomicrobia has recently emerged as a key phylum in both terrestrial and aquatic environments. Just as previous soil research has identified this group as prevalent and highly abundant [7], our study highlights this phylum’s high relative abundance and ubiquity across 14 freshwater lakes. While class-level habitat preferences were identified, variation in Verrucomicrobia relative abundance and community composition was poorly explained by the measured geochemical parameters, indicating that additional abiotic and biotic interactions are involved. One speculated factor is the abundance and nature of organic matter, as various studies have identified Verrucomicrobia genomic features involved in diverse carbon metabolism. Given their potential to act as important players in carbon cycling, and the importance of freshwater carbon cycling for global carbon fluxes [28, 30], further research is needed to examine Verrucomicrobia functional roles and ecophysiology in freshwater ecosystems.

## Materials and methods

### Field sampling

We collected samples from 12 inland lakes located in Southeastern Michigan, Lake Michigan, and one of its freshwater estuaries, the drowned river mouth lake Muskegon Lake. Sample sites are illustrated in Fig 1 using GPS coordinates (S1 Table) and the U.S. Geological Survey, National Geospatial Program, National Map Viewer in basemap “Light Gray Canvas.” As no animal species were collected, and the lakes were on public lands, no specific permissions were required for these locations/activities, and the field studies did not involve endangered or protected species.

#### Lake Michigan survey

Replicates of two fractions (FL: 0.22–3 um; PA: 3–20 um) from 18 distinct samples from Lake Michigan and one Muskegon Lake site (buoy) were collected on April 23–24, July 15–16, and September 23–24, 2013 using a procedure highly similar to the one described below for Muskegon Lake and inland lake samples, and detailed previously [50]. Limnological characterization of the water samples from Lake Michigan included Chlorophyll *a* (Chl *a*), dissolved oxygen (DO), fluorescence, dissolved organic carbon (DOC), particulate organic carbon (POC), photosynthetically active radiation (PAR), particulate organic nitrogen (PON), particulate phosphorus (PP), total phosphorus (TP), silicon dioxide (SiO_2_), temperature, and total suspended solids (TSS). Detailed methods were described previously [51].

#### Muskegon Lake survey

Replicates of PA and FL fractions of 22 distinct Muskegon Lake water samples as well as 11 sediment samples were collected on May 13, July 22, and September 24, 2014 in collaboration with the Grand Valley State University’s Annis Water Resources Institute. Samples in May were collected at three sites (Inlet, Outlet, Deep) while in July and September, samples were collected at an additional site: Bear (Fig 1). At each site, duplicate samples were taken 0.2–0.6 m below the surface, 0.9–3.9 m above the bottom, and of the sediment. Water samples were collected with a Van Dorn bottle and immediately prefiltered through a 210 μm and 20 μm nitex cloth (WildCo., Yulee, FL). For water samples intended for DNA extraction, the filtrate was filtered through a 3 μm isopore membrane filter (TSTP, 47 mm diameter, Millipore, Billerica, MA) to collect the PA fraction (20–3 μm), and a 0.22 μm express plus membrane filter (47 mm diameter, Millipore, Billerica, MA) to collect the FL fraction (3–0.22 μm). Sequential in-line filtration was performed using an easy-load L/S/ peristaltic pump head (Masterflex®, Cole Palmer Instrument Company, Vernon Hills, IL) with a 47 mm polycarbonate in-line filter holder (Pall Corporation, Ann Arbor, MI). After filtration, the filters were folded with the biomass side facing in, placed in 2 mL cryovials with RNA*later*® (Life Technologies, Carlsbad, CA) within 32 minutes. Sediment samples were collected with a Ponar grab and placed in 2 mL cryovials. All samples were stored in liquid nitrogen on board and during transport, and in our laboratory at -80°C until DNA extraction. Limnological characterization of Muskegon Lake samples included: (a) temperature, pH, nitrate, ammonia, soluble reactive phosphorus (SRP), TP, DO, Chl *a*, and turbidity as described in [40], (b) total dissolved solids, oxidation-reduction potential and blue-green algae measured using a YSI 6600 V2-4 Multi-Parameter Water Quality Sonde, and (c) chloride, sulfate, total kjeldahl nitrogen, and alkalinity measurements according to US EPA Rev 18 methods [40].

#### Inland lakes survey

PA and FL fractions of 64 distinct inland lake samples were collected from 12 lakes (Big Seven, Bishop, Bruin, Cedar, Halfmoon, Heron, Independence, Lobdell, North, Sand, Whitmore, Woodland) in June 2014 across a three point transect (nearshore, deepest point of lake, nearshore). At each point, water was sampled 1 m below the surface. At the deepest point of the lake, an additional sample 1–2 m above the bottom of the lake was collected. In Oct 2014 and Apr 2015, samples were collected from 4 of the inland lakes (Bruin, Independence, North, Whitmore). Water was collected at the deepest point of the lake, 1 m below the surface, and above the bottom of the lake. Samples were analyzed for TP, total dissolved P, SRP, nitrate, ammonium, and Chl *a* as previously described [52].

### DNA extraction

All water samples were extracted using an optimized protocol previously described [17], but the extraction kit differed between samples. All Lake Michigan survey samples and Muskegon Lake survey water samples from May had DNA extracted using the AllPrep DNA/RNA Kit (Qiagen, Venlo, The Netherlands). Inland lake samples and Muskegon Lake survey water samples from June and September had DNA extracted using a modified version of the DNeasy Blood & Tissue Kit (Qiagen). This protocol used the same buffers and sequence of operations as in the DNA extraction portion of the AllPrep DNA/RNA Kit in order to eliminate extraction protocol differences between the different sample sets. Prior to extraction, filters were cut in half, dipped into PBS to remove excess RNA*later*, blotted on Kimwipes (Kimtech, Irving, TX), and placed into 2 mL tubes. Prepared filters that were not immediately extracted were stored at -20°C and processed within 3 days as previously described [17]. DNA extracts were stored at 4°C until sequencing. DNA concentration was quantified using the Quant-iT™ Picogreen® dsDNA Assay Kit (Life Technologies).As the Qiagen kit was not optimized for high organic matter-containing samples, we extracted sediment DNA using the PowerSoil® DNA Isolation Kit (MO BIO Laboratories, Inc., Carlsbad, CA) according to the manufacturer’s instructions.

### DNA sequencing and processing

Lake Michigan survey DNA extractions were submitted for sequencing by the DOE Joint Genome Institute (JGI). The 16S amplification part of the primers used by the JGI (515F/806R) are the same as used in the protocol described below; however, the remainder of the primers differs [53, 54], which may affect amplification bias. The Muskegon Lake survey DNA extractions from May samples were submitted for sequencing on June 13, 2014, while the remaining Muskegon Lake and inland lake samples were submitted on Feb 25, 2015; both sequencing runs were performed at the Microbial Systems Laboratories at the University of Michigan Medical School. Illumina MiSeq v2 chemistry 2x250 (500 cycles) was performed using dual index-labeled primers that target the V4 region of the 16S rRNA (515F/806R) according to [53]. Data was processed with mothur v. 1.34.3 [55] following the MiSeq standard operating procedure (accessed on Mar 13, 2015). The Silva database (release 119) was used for sequence alignment and classification using the Wang method, which performs Bayesian classification of 8-base kmers [56]. Mothur outputs were combined in R version 3.2.3 [56] using the phyloseq package [57]. Non-bacterial and chloroplast sequences were removed before duplicates were combined and averaged to create a dataset of only unique samples. Two samples with fewer than 2,000 reads were removed. Data was normalized by multiplying the relative sequence abundance by the smallest library size (2,072 sequences) and rounding the result in order to account for heteroscedasticity in sequencing depth [57]. All data visualization used the *ggplot2* package [58] unless noted otherwise. Fastq files from the Lake Michigan survey are available at the Joint Genome Institute’s genome data portal (https://genome.jgi.doe.gov/; Project IDs = 1041195 and 1041198). Fastq files from the Muskegon Lake survey were submitted to the NCBI sequence read archive under BioProject PRJNA412983. Fastq files from the inland lake survey were submitted to NCBI sequence read archive under BioProject PRJNA414423. All code to replicate our analyses and generate the figures is available at https://github.com/DenefLab/Verruco/.

### Verrucomicrobia relative abundance

Relative abundance of Verrucomicrobia was compared using Kruskal-Wallis and post-hoc tests (*kruskal*.*test*, *stats* package [56]; *kruskalmc*, *pgirmess* package [59]), and p-values were adjusted for false discovery rate using the Benjamini-Hochberg procedure (*p*.*adjust*, *stats* package [56]). Except for the analysis in S1 Fig that examines the effect on relative abundance due to difference in sequencing procedures at UM and the JGI, Lake Michigan survey samples from Muskegon Lake (buoy station) were removed to avoid introducing bias. Drivers of Verrucomicrobia relative abundance were determined by evaluating the best multiple linear regression model. To minimize bias, we evaluated co-correlation between environmental variables and removed those that exhibited correlation (*pairs*, *graphics* package [56]). Next, we log-transformed environmental data and Verrucomicrobia abundance to improve linearity when plotted against each other (*xyplot*, *lattice* package [60]; *lm*, *stats* package [56]). To minimize bias due to non-linear data, we removed variables that were not linear. *graphics* package [56]). Only the remaining variables, which were linear and uncorrelated with other variables, were included in generating the model with the lowest Schwartz’s Bayesian information criterion (exhaustive search to examine all possible combinations of environmental data) (*regsubsets*, *leaps* package; *lm [61]*). Models were evaluated separately for each lake type due to differences in the available environmental data.

### Within-phylum diversity

The inverse Simpson index was calculated by sampling sequences with replacement to the smallest number of reads (2,072), and averaging over 100 trials (*estimate_richness*, *phyloseq* package [57]). Kruskal-Wallis tests were performed as described above to determine significance between inverse Simpson of different samples.

### Phylogenetic diversity

The Verrucomicrobia phylogenetic tree was created in mothur using *clearcut* (relaxed neighbor joining algorithm) using uncorrected pairwise distance between aligned sequences (*dist*.*seqs*)) after extracting representative sequences for 1,323 OTUs using *get*.*oturep*. The tree was created separately from the Silva database template taxonomy tree used to classify sequences, hence sequences designated as unclassified by the classification algorithm may appear clustered with classified sequences in this tree. To determine phylogenetic relatedness of verrucomicrobial communities, standardized effect size mean pairwise distance (SES MPD; [62]) was calculated by comparing Faith’s index to a null model over 999 iterations. (*ses*.*mpd*, *picante* package [63]). While Faith’s index is positively correlated with richness, SES MPD is a measure of phylogenetic diversity that is unbiased by richness. Positive SES MPD values indicate more phylogenetic evenness compared to the null model, while negative SES MPD values indicate more phylogenetic clustering.

### Verrucomicrobia and non-Verrucomicrobia community composition

Bray-Curtis dissimilarity was calculated (*distance*, *phyloseq* package [57]) and used to create Principal Coordinate Analysis (PCoA) plots (*ordinate*, *phyloseq* package) for both Verrucomicrobia and non-Verrucomicrobia bacterial communities. To evaluate significance between dissimilarity matrices, we performed Permutational Multivariate Analysis of Variance (PERMANOVA) (*adonis*, *vegan* package [64]). Procrustes analyses were performed to determine the correlation between Verrucomicrobia and non-Verrucomicrobia communities by comparing the spatial composition of their respective PcoA ordinations (*procrustes* and *protest*, *vegan* package; [65]). Bioenv analyses were performed to determine the environmental drivers of Verrucomicrobia and non-Verrucomicrobia community compositions by calculating the highest Spearman correlation between similarity matrices (*bioenv*, *vegan* package). All available environmental data were included in the analyses, as bioenv is not constrained by data distribution. The analyses were performed separately for each lake type due to differences in the available environmental data.

### Class-level habitat specialization within Verrucomicrobia

To evaluate phylogenetic distinctness of different samples, we calculated weighted UniFrac (*distance*, *phyloseq* package [57]) and determined significance using PERMANOVA (*adonis*, *vegan* package [64]). Further analyses were performed at the class level as this was the most resolved taxonomic level for the majority of Verrucomicrobia (93.4%). To identify differential relative abundances of specific Verrucomicrobia classes, we used the *DESeq2* package [66], which uses count data to estimate variance-mean dependence in order to evaluate differential relative abundance based on the negative binomial distribution. This was performed on pairwise comparisons within each habitat category (Depth, Lake Type, Fraction, Season). For example, to determine season preference for spring, two comparisons were performed: spring versus summer, and spring versus fall. The intersect of OTUs that were significantly more abundant in spring from both comparisons was then selected as those which had a preference for spring. Significance of DESeq results was determined by calculating Pagel’s λ (*geiger* package [67]). DESeq results were visualized alongside the Verrucomicrobia tree using Interactive Tree of Life [68] and tree bootstrap values were calculated using the *phangorn* package [69].

## Supporting information

S1 Table Limnological data for samples included in the linear modelling and bioenv analyses. **Table is organized by A) Laurentian survey samples, B) estuary survey samples, and C) inland lake survey samples.** BGA = blue-green algae, Chl *a* = Chlorophyll *a*, DO = dissolved oxygen, DOC = dissolved organic carbon, NH_3_ = ammonia, NH4 = ammonium, NO_3_ = nitrate, ORP = oxidation-reduction potential, PAR = photosynthetically active radiation, POC = particulate organic carbon, PON = particulate organic nitrogen, PP = particulate phosphorus, SiO_2_ = silica, SO_4_ = sulfate, SRP = soluble reactive phosphorus, TDP = total dissolved phosphorus, TKN = total Kjeldahl nitrogen, TP = total phosphorus, TSS = total suspended solids.
(PDF)

S1 Fig Comparison of Laurentian and estuary survey samples. Relative abundance of Verrucomicrobia in samples collected during (A) Laurentian survey and (B) in all estuary water samples. Red plus signs indicate significance of one subset of samples within the panel. A letter indicates significance between two sample categories within the panel.
(PDF)

S2 Fig Whole bacterial community phylum-level relative abundance. Samples are categorized by lake type (horizontal), fraction (vertical), and season (vertical). Error bars represent the interquartile range.
(PDF)

S3 Fig Multiple linear model residual plots. Residual plots for the best multiple linear model for (A) Laurentian, (B) estuary, and (C) inland samples.
(PDF)

S4 Fig Verrucomicrobia phylogenetic diversity. Phylogenetic diversity measured by standardized effect size mean pairwise distance (SES MPD) (bottom). Samples are categorized by (A) lake type, (B) fraction, and (C) season. Red asterisks indicate significance between all samples in the panel. Red plus signs indicate significance of one subset of samples within the panel. A letter indicates significance between two sample categories within the panel.
(PDF)

S5 Fig Verrucomicrobia phylogenetic tree with bootstrap values. Verrucomicrobia phylogenetic tree consists of OTUs with a significant preference in at least one habitat. Bootstrap values above 50 are displayed, and known OTU taxonomic classifications are shown.
(PDF)

S6 Fig Verrucomicrobia within-phylum relative abundance. Samples are categorized by lake type (horizontal), fraction (vertical), and season (vertical). Error bars represent the interquartile range.
(PDF)
