# Correction: Verrucomicrobia are prevalent in north-temperate freshwater lakes and display class-level preferences between lake habitats

**DOI:** 10.1371/journal.pone.0206396

**Published:** 2018-10-22

**Authors:** Edna Chiang, Marian L. Schmidt, Michelle A. Berry, Bopaiah A. Biddanda, Ashley Burtner, Thomas H. Johengen, Danna Palladino, Vincent J. Denef

Fig 3 is incorrect. The symbols for “Free” and “Sediment” under the “Fraction” section of the legend are incorrect. The authors have provided a corrected version here.

**Fig 3:**
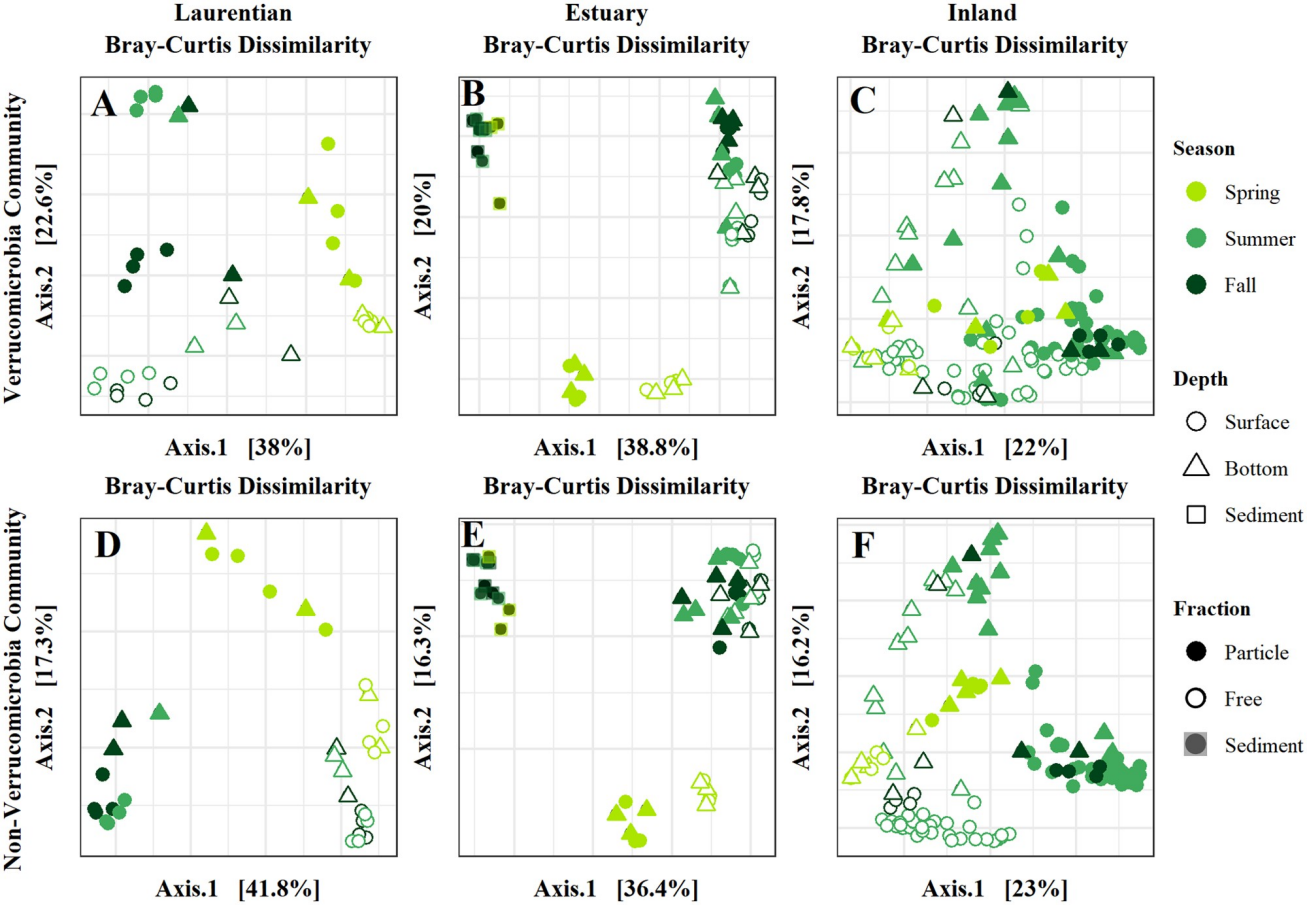
Principal coordinates analysis (PCoA) ordinations (first two principal coordinates are displayed) based on Bray-Curtis dissimilarity. PCoAs visualizing the compositional differences of (A-C) the verrucomicrobial and (D-F) the whole bacterial community in Laurentian, estuary, and inland lake samples, respectively. Data points are colored by season, shaped by depth, and filled in by fraction. Axis labels include the % variation captured by the respective dimension of the ordination.

## References

[1] Chiang E, Schmidt ML, Berry MA, Biddanda BA, Burtner A, Johengen TH, et al (2018) Verrucomicrobia are prevalent in north-temperate freshwater lakes and display class-level preferences between lake habitats. PLoS ONE 13(3): e0195112 10.1371/journal.pone.0195112 29590198 PMC5874073

