# Supplementary material for: Verrucomicrobia are prevalent in north-temperate freshwater lakes and display class-level preferences between lake habitats
Source: PLoS One. 2018 Mar 28;13(3):e0195112. doi: 10.1371/journal.pone.0195112 (PMC5874073; doi:10.1371/journal.pone.0195112)
Supplement: S1 Table — Table is organized by A) Laurentian survey samples, B) estuary survey samples, and C) inland lake survey samples. BGA = blue-green algae, Chl a = Chlorophyll a, DO = dissolved oxygen, DOC = dissolved organic carbon, NH3 = ammonia, NH4 = ammonium, NO3 = nitrate, ORP = oxidation-reduction potential, PAR = photosynthetically active radiation, POC = particulate organic carbon, PON = particulate organic nitrogen, PP = particulate phosphorus, SiO2 = silica, SO4 = sulfate, SRP = soluble reactive phosphorus, TDP = total dissolved phosphorus, TKN = total Kjeldahl nitrogen, TP = total phosphorus, TSS = total suspended solids. (PDF) [file pone.0195112.s001.pdf]

Supporting Information

S1 Table. Limnological data for samples included in the linear modelling and bioenv analyses. Table is organized by A) Laurentian survey samples, B) estuary survey samples, C) inland lake survey samples.

BGA = blue-green algae, Chl *a* = Chlorophyll *a*, DO = dissolved oxygen, DOC = dissolved organic carbon, NH_3 = ammonia, NH_4 = ammonium, NO_3 = nitrate, ORP = oxidation-reduction potential, PAR = photosynthetically active radiation, POC = particulate organic carbon, PON = particulate organic nitrogen, PP = particulate phosphorus, SiO_2 = silica, SO_4 = sulfate, SRP = soluble reactive phosphorus, TDP = total dissolved phosphorus, TKN = total Kjeldahl nitrogen, TP = total phosphorus, TSS = total suspended solids

A) Laurentian Survey Samples

Station	GPS coordinates north	GPS coordinates west	Depth	Sample Size	Sample Depth (m)	Temperature (C)	DO (mg/L)	Chl (ug/L)	TP (ug/L)	PP (ug/L)	TDP (ug/L)	POC (mg/L)	PON (mg/L)	DOC (mg/L)	SiO2 (mg/L)	TSS (mg/L)	Fluorescence (mg/m3)	PAR
Nearshore	43.18806	86.34389	Surface	11	5	12.36	12.38	2.90	11.23	8.57	2.66	0.39	0.05	3.98	1.63	2.59	1.37	247.89
Offshore	43.19972	86.56972	Surface	10	5	13.21	11.27	0.92	4.35	2.46	1.89	0.20	0.03	2.32	1.46	0.39	0.44	32.15
			Bottom	3	105	4.27	13.78	0.19	2.70	1.06	1.64	0.07	0.01	1.94	1.91	0.10	0.07	0.00
Buoy	43.240965	86.278965	Surface	9	1.5	23.303	8.63857	NA	NA	NA	NA	NA	NA	NA	NA	NA	14.23325	522.648
			Bottom	6	9.2	17.317	0	NA	NA	NA	NA	NA	NA	NA	NA	NA	13.11175	0.02155

B) Estuary Survey Samples

Station	GPS coordinates north	GPS coordinates west	Depth	Sample Size	Sample Depth (m)	Temperature (C)	TDS (mg/L)	pH	ORP (mV)	Chl (ug/L)	BGA (cells/mL)	Cl (mg/L)	SO4 (mg/L)	NO3 (mg/L)	NH3 (mg/L)	TKN (mg/L)	SRP (ug/L)	TP (ug/L)	Alk (mg/L)	DO (mg/L)	Turbidity (NTU)
Inlet	43.25208	86.25242	Surface	6	1	19.76	265.00	8.14	404.67	4.35	4624.33	20.67	15.00	0.80	0.02	0.53	2.50	23.27	141.33	9.22	5.83
			Bottom	6	6.82	19.46	266.33	8.09	404.33	2.23	2159.33	21.33	15.33	0.79	0.02	0.48	2.50	24.57	147.67	8.69	6.13
Bear	43.2386	86.29917	Surface	4	1	20.46	246.00	8.17	404.00	2.94	3882.50	24.50	16.00	0.79	0.04	0.55	2.50	20.35	151.00	9.31	3.55
			Bottom	4	15.61	11.34	216.00	7.39	354.00	0.29	1478.50	23.50	19.50	0.85	0.08	0.30	2.50	19.25	135.00	6.45	4.30
Outlet	43.23297	86.32684	Surface	6	1	18.80	222.33	8.03	409.00	2.73	9001.33	23.33	14.67	0.71	0.02	0.54	2.50	20.20	139.33	9.28	3.73
			Bottom	6	9.57	17.19	223.00	7.80	412.00	0.96	1298.33	23.00	14.67	0.51	0.04	0.59	4.00	18.53	136.00	7.79	2.77
Deep	43.22306	86.29809	Surface	6	1	18.69	224.33	7.99	421.00	2.52	58089.33	22.00	14.67	0.75	0.02	0.63	2.50	18.27	137.33	9.31	3.57
			Bottom	6	22.87	12.36	203.00	7.44	360.33	0.54	1220.67	18.67	16.67	0.81	0.03	0.46	13.73	34.27	125.67	5.66	0.80

C) Inland Lake Survey Samples

Cedar	GPS coordinates north	GPS coordinates west	Total Depth (ft)	Depth	Sample Size	Sample Depth (m)	Chl (ug/L)	NO3 (mg/L)	SRP (ug/L)	TP (ug/L)	TDP (ug/L)	NH4 (ug/L)
**Big Seven**	42.81163	83.67762	51.5	Surface	6	2	4.64	0.16	0.14	17.26	4.47	15.46
				Bottom	2	13	5.28	0.16	17.01	48.19	17.29	730.43
**Bishop**	42.50139	83.83851	56.0	Surface	6	2	4.64	0.17	14.05	14.88	4.39	9.72
				Bottom	2	14	7.63	0.16	19.23	60.16	16.70	1056.53
**Bruin**	42.41941	84.03976	49.8	Surface	10	2	1.81	0.15	4.43	8.19	3.53	40.20
				Bottom	6	11.8	2.90	0.14	9.41	12.44	3.59	440.43
**Cedar**	42.31501	84.08047	24.6	Surface	6	2	3.81	0.17	0.64	13.72	4.81	80.46
				Bottom	2	5	4.95	0.17	8.38	18.94	5.13	216.38
**Halfmoon**	42.42014	84.01286	88.0	Surface	6	2	7.84	0.20	0.78	19.79	8.28	7.39
				Bottom	2	23	0.57	0.29	144.14	116.59	111.21	296.23
**Heron**	42.8088	83.52392	42.9	Surface	6	2	4.14	0.16	0.16	16.58	3.76	7.05
				Bottom	2	10	17.04	0.17	0.17	55.10	4.41	588.12
**Independence**	42.40535	83.80415	36.0	Surface	10	2	3.65	0.13	0.78	12.90	4.96	75.20
				Bottom	6	7.1	3.21	0.11	0.96	12.43	4.27	112.23
**Lobdell**	42.78851	83.83338	83.0	Surface	6	2	3.34	0.17	0.95	14.40	5.94	4.84
				Bottom	2	20	0.39	0.21	60.18	54.69	44.68	116.47
**North**	42.39342	84.00815	52.2	Surface	10	2	6.56	0.14	2.72	13.69	4.52	4.74
				Bottom	4	12.5	10.17	0.10	1.26	48.81	6.38	283.12
**Sand**	42.04443	84.1368	37.2	Surface	6	2	1.83	1.83	1.02	9.88	3.27	3.48
				Bottom	2	9	3.47	3.47	1.15	11.66	3.74	81.48
**Whitmore**	42.43639	83.74645	61.0	Surface	10	2	3.60	0.15	8.67	15.93	3.69	15.09
				Bottom	6	14.6	1.75	0.14	0.04	14.07	3.61	83.02
**Woodland**	42.55198	83.77474	32.6		6	2	2.70	0.16	0.26	14.34	5.24	7.67
				Bottom	2	8	30.90	0.16	0.00	62.56	5.72	300.30
