# Supplementary material for: Verrucomicrobia are prevalent in north-temperate freshwater lakes and display class-level preferences between lake habitats
Source: PLoS One. 2018 Mar 28;13(3):e0195112. doi: 10.1371/journal.pone.0195112 (PMC5874073; doi:10.1371/journal.pone.0195112)

S1 Fig. Comparison of Laurentian and estuary survey samples. Relative abundance of Verrucomicrobia in samples collected during (A) Laurentian survey and (B) in all estuary water samples. Red plus signs indicate significant of one subset of samples within the panel. A letter indicates significant between two sample categories within the panel.

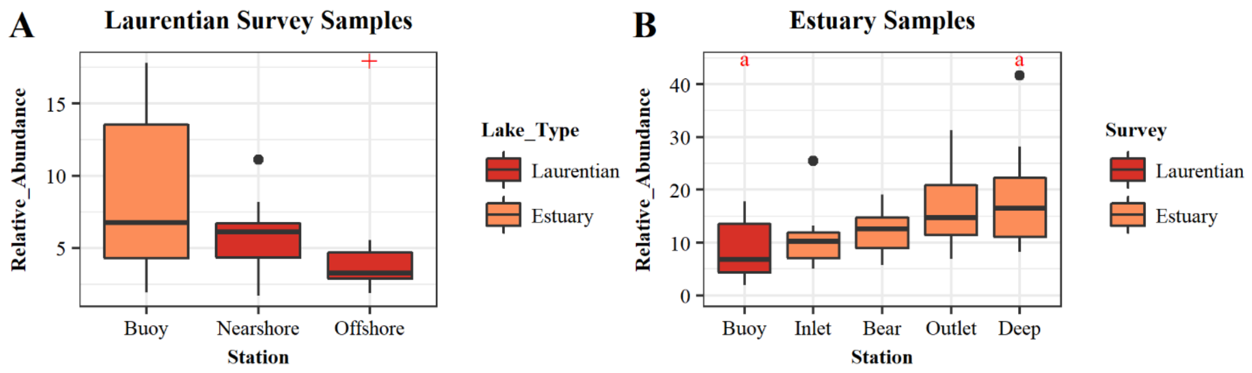

Supplement: S1 Fig — Relative abundance of Verrucomicrobia in samples collected during (A) Laurentian survey and (B) in all estuary water samples. Red plus signs indicate significance of one subset of samples within the panel. A letter indicates significance between two sample categories within the panel. (PDF) [file pone.0195112.s002.pdf]
