# Supplementary material for: Verrucomicrobia are prevalent in north-temperate freshwater lakes and display class-level preferences between lake habitats
Source: PLoS One. 2018 Mar 28;13(3):e0195112. doi: 10.1371/journal.pone.0195112 (PMC5874073; doi:10.1371/journal.pone.0195112)

S2 Fig. Whole bacterial community phylum-level relative abundance. Samples are categorized by lake type (horizontal), fraction (vertical), and season (vertical). Error bars represent the interquartile range.

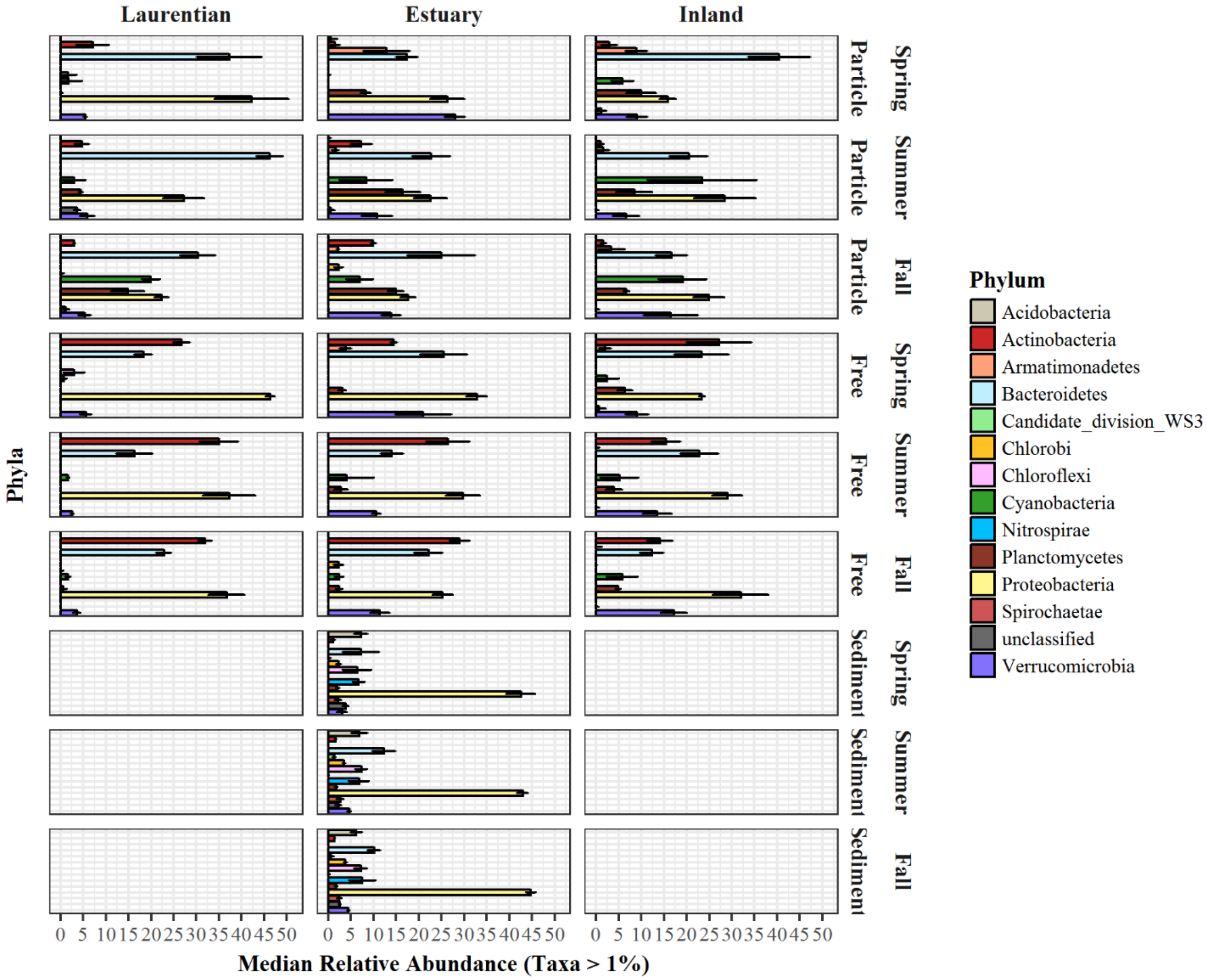

Supplement: S2 Fig — Samples are categorized by lake type (horizontal), fraction (vertical), and season (vertical). Error bars represent the interquartile range. (PDF) [file pone.0195112.s003.pdf]
