# Supplementary material for: Verrucomicrobia are prevalent in north-temperate freshwater lakes and display class-level preferences between lake habitats
Source: PLoS One. 2018 Mar 28;13(3):e0195112. doi: 10.1371/journal.pone.0195112 (PMC5874073; doi:10.1371/journal.pone.0195112)

S3 Fig. Multiple linear model residual plots. Residual plots for the best multiple linear model for (A) Laurentian, (B) estuary, and (C) inland samples.

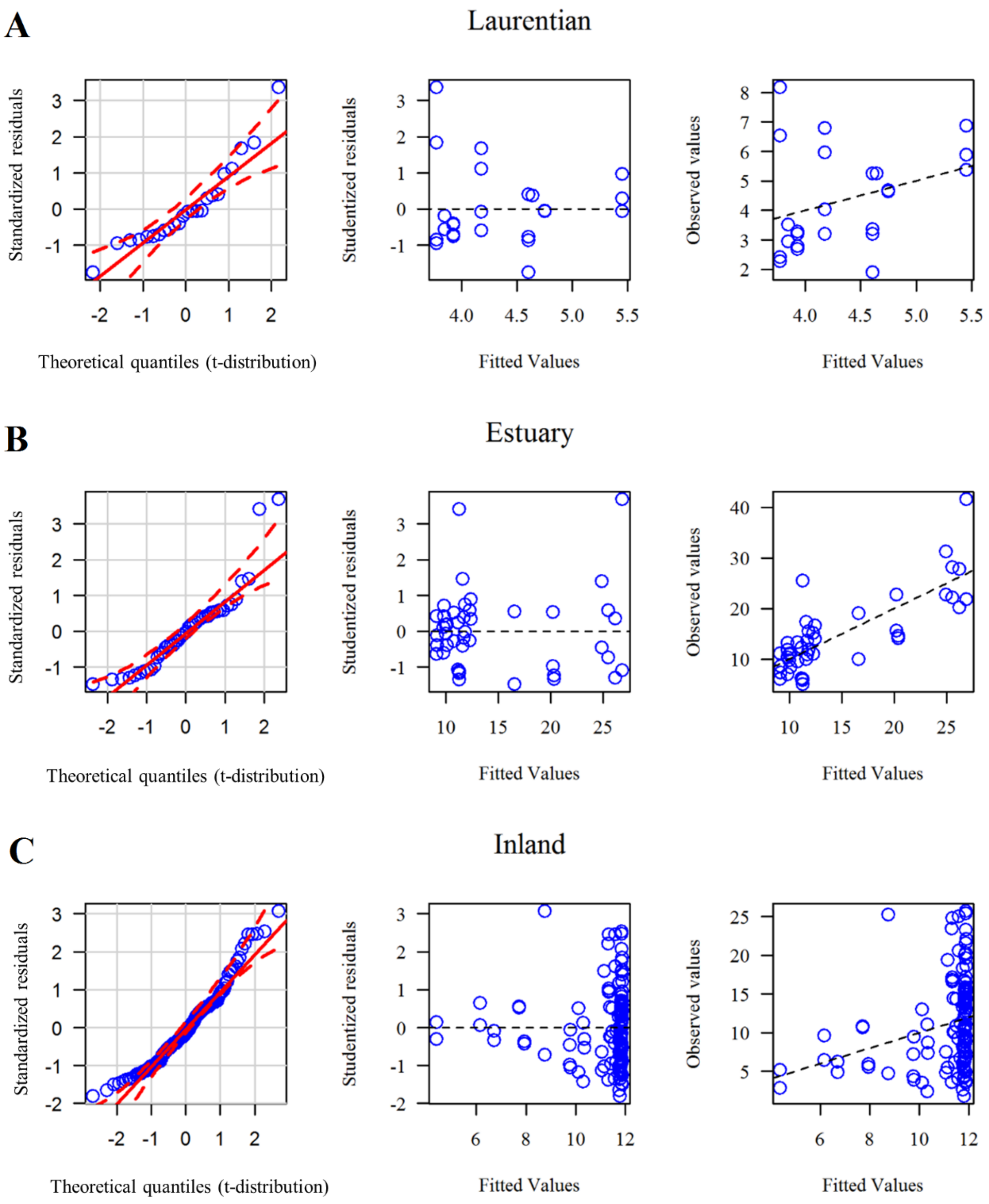

Supplement: S3 Fig — Residual plots for the best multiple linear model for (A) Laurentian, (B) estuary, and (C) inland samples. (PDF) [file pone.0195112.s004.pdf]
