# Supplementary material for: Verrucomicrobia are prevalent in north-temperate freshwater lakes and display class-level preferences between lake habitats
Source: PLoS One. 2018 Mar 28;13(3):e0195112. doi: 10.1371/journal.pone.0195112 (PMC5874073; doi:10.1371/journal.pone.0195112)

S4 Fig. Verrucomicrobia phylogenetic diversity. Phylogenetic diversity measured by standardized effect size mean pairwise distance (SES MPD). Samples are categorized by (A) lake type, (B) fraction, and (C) season. Red asterisks indicate significant between all samples in the panel. Red plus signs indicate significant of one subset of samples within the panel. A letter indicates significant between two sample categories within the panel.

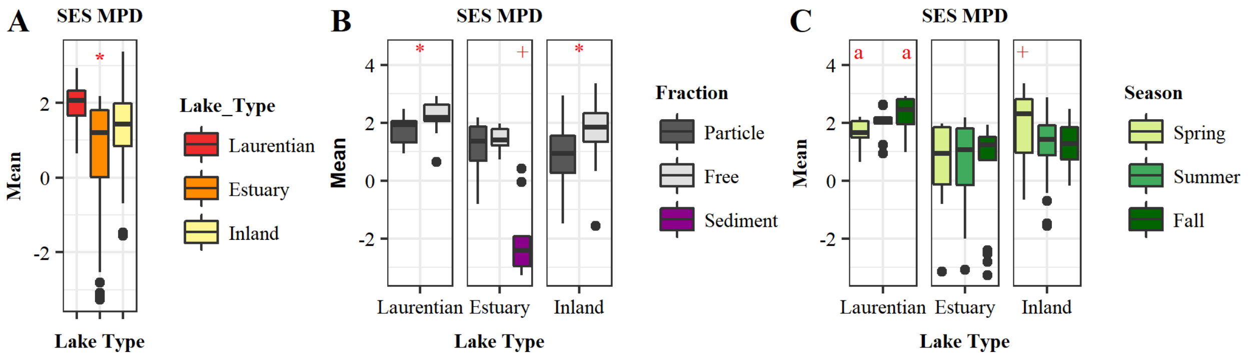

Supplement: S4 Fig — Phylogenetic diversity measured by standardized effect size mean pairwise distance (SES MPD) (bottom). Samples are categorized by (A) lake type, (B) fraction, and (C) season. Red asterisks indicate significance between all samples in the panel. Red plus signs indicate significance of one subset of samples within the panel. A letter indicates significance between two sample categories within the panel. (PDF) [file pone.0195112.s005.pdf]
