# Supplementary figures and images for: Verrucomicrobia are prevalent in north-temperate freshwater lakes and display class-level preferences between lake habitats
Source: PLoS One. 2018 Mar 28;13(3):e0195112. doi: 10.1371/journal.pone.0195112 (PMC5874073; doi:10.1371/journal.pone.0195112)

5

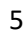

Supplement: S5 Fig — Verrucomicrobia phylogenetic tree consists of OTUs with a significant preference in at least one habitat. Bootstrap values above 50 are displayed, and known OTU taxonomic classifications are shown. (PDF) [file pone.0195112.s006.pdf]
